# Synthetic Small Molecules as Regulators of In Vitro Multiplication in *Selenicereus* Hybrids

**DOI:** 10.3390/plants15131931

**Published:** 2026-06-23

**Authors:** Malen Escánez, Alejandro Miralles-Rodríguez, Sandra Gil, Francisco Bermúdez, Santiago Vilanova, Elena Carneros, Ana Martinez, Carmen Gil, Pilar S. Testillano, Edgar García-Fortea

**Affiliations:** 1 Seeds for Innovation S.L., Edificio PITA, Lab 43, Carretera Sacramento s/n, 04120 Almeria, Spain; alejandro.miralles@beyond-seeds.com (A.M.-R.); sandra.gil@beyond-seeds.com (S.G.); francisco.bermudez@beyond-seeds.com (F.B.); edgar.garcia@beyond-seeds.com (E.G.-F.); 2 Instituto de Conservación y Mejora de la Agrodiversidad Valenciana, Universitat Politècnica de València, 46022 Valencia, Spain; sanvina@upvnet.upv.es; 3 Pollen Biotechnology of Crop Plants Group, Margarita Salas Center for Biological Research (CIB), Spanish National Research Council (CSIC), Ramiro de Maeztu 9, 28040 Madrid, Spain; ecarneros@cib.csic.es (E.C.); testillano@cib.csic.es (P.S.T.); 4 Translational Medicinal and Biological Chemistry Group, Margarita Salas Center for Biological Research (CIB), Spanish National Research Council (CSIC), Ramiro de Maeztu 9, 28040 Madrid, Spain; ana.martinez@csic.es (A.M.); carmen.gil@csic.es (C.G.)

**Keywords:** *Selenicereus*, pitahaya, small molecule inhibitors, micropropagation, cell reprogramming

## Abstract

Micropropagation of *Selenicereus* hybrids is a key tool for breeding and conservation; however, further refining the balance between high multiplication rates and morphological quality remains a complex challenge within conventional protocols. This study explores targeted signaling modulation using nine bioactive small molecules—including three mammalian glycogen synthase kinase 3 (GSK3) inhibitors (TDZD-9, VP3.15 and VP0.7), three leucine rich repeat kinase 2 (LRRK2) inhibitors (JZ1.24, JZ1.3 and IGS4.75), and three phosphodiesterase (PDE) inhibitors—to complement traditional micropropagation. Explants were evaluated in two distinct contexts: a hormone-free basal medium (BM) and a plant growth regulator-supplemented medium (PIT2) and the response rates, yield, and quality were measured and integrated using a Global Efficiency Index (GEI). Results demonstrate that inhibitor efficacy is strictly context-dependent; while most molecules repressed budding in BM, they acted as response modulators by determining the specific type of morphogenic pathway in PIT2. Notably, the GSK3 inhibitor TDZD-9 reached the highest GEI (0.85) by maximizing productivity, whereas LRRK2 inhibitors effectively preserved architectural integrity. Flow cytometry confirmed cytogenetic stability across all treatments, with a 98.5% plantlet survival rate during acclimatization. In conclusion, the strategic integration of targeted signaling modulators and multi-parametric indices offers a refined and objective framework to enhance the efficiency of mass propagation protocols in pitahaya and other recalcitrant species. Furthermore, our findings provide new evidence of the strong potential of these small molecules as novel tools to improve plant micropropagation beyond traditional plant growth regulators.

## 1. Introduction

In recent years, pitahaya (*Selenicereus* spp., formerly *Hylocereus*) has attracted increasing attention as an emerging fruit crop in Mediterranean regions due to its adaptability to semi-arid environments and its potential contribution to crop diversification under water-limited agricultural conditions [1]. As its cultivation expands beyond traditional production areas, the development of efficient propagation systems has become imperative. Due to the predominantly outcrossing nature of these species and the resulting high heterozygosity, seed propagation is generally restricted to breeding programs, as it does not guarantee the preservation of elite traits. While vegetative propagation through cuttings is the standard commercial practice, it carries the risk of pathogen dissemination and limits the availability of certified material. Consequently, micropropagation has emerged as a vital strategy for the large-scale production of uniform, pathogen-free, and cost-effective elite genotypes [2,3].

In this context, significant advances in micropropagation have been achieved for species such as *S. undatus* and *S. costaricensis* using various explants, including cladode segments and areoles. These protocols typically rely on combinations of cytokinins like 6-benzylaminopurine (6-BAP) or zeatin and auxins like indole-3-butyric acid (IBA) to promote shoot proliferation and elongation, frequently achieving high regeneration rates and successful acclimatization [2,3,4]. More recently, the implementation of temporary immersion systems has enabled improvements in large-scale multiplication while enhancing growth performance [5], while studies on photomixotrophic capacity have established a robust methodological framework for pitahaya [6]. Despite these advances, regeneration efficiency remains strongly genotype-dependent. Interspecific hybrids frequently display variable responses, often requiring recurrent empirical adjustments to hormonal ratios [7]. In this regard, *Selenicereus* hybrids represent a versatile experimental framework to explore the fundamental mechanisms of plant morphogenesis. Given its predictable response to exogenous plant growth regulators (PGRs), pitahaya serves as a convenient model system to investigate the morphogenic potential of non-canonical signaling modulators in a micropropagation context.

Traditionally, protocols have focused on modifying the exogenous auxin–cytokinin balance [8]. Nevertheless, emerging strategies are shifting toward the modulation of endogenous biosynthesis pathways and epigenetic reprogramming to sensitize tissues and change the cell fate through biochemical modulators distinct from those traditional regulators [9]. This study moves away from empirical hormonal adjustment to conduct a preliminary phenotypic screening exploring small-molecule inhibitors originally characterized in mammalian systems. These compounds have shown significant results in stress-induced cell reprogramming protocols in several model crop and forest species, such as *Brassica napus* and *Hordeum vulgare* microspore cultures and *Quercus suber* somatic embryogenesis. Among them, inhibitors of Glycogen Synthase Kinase 3 (GSK3), Leucine-Rich Repeat Kinase 2 (LRRK2), and phosphodiesterases (PDEs) are particularly promising [10,11,12].

In plants, GSK3 orthologs, such as Brassinosteroid-Insensitive 2 (BIN2), act as master negative regulators of the brassinosteroid (BR) signaling pathway. In the absence of sufficient BR levels, these kinases remain active, phosphorylating key transcription factors to suppress growth-related gene expression. Pharmacological inhibition of GSK3 has been proposed as a chemical genetics strategy to bypass this repression, potentially mimicking a constitutive BR response even in recalcitrant genotypes [13]. This modulation is of particular interest in micropropagation, as BR signaling is essential for maintaining meristematic architecture and governing the microtubule organization required for proper cell elongation and the control of apical dominance [14,15]. In *B. napus* microspore cultures, treatments with GSK3 inhibitors increased microspore embryogenesis efficiency while they activated the BR signaling pathway [11].

In parallel, an innovative approach involves the use of LRRK2 inhibitors. While its roles in mammals are still being elucidated, LRRK2 is recognized as a critical scaffolding protein within *Wnt* signaling cascades [16]. Despite the absence of strict LRRK2 orthologs in plants, the fundamental mechanisms governing stem cell behavior show striking functional similarities across kingdoms [17]. Specifically, recent evidence across diverse embryogenesis models (including herbaceous and woody species) as well as in *Arabidopsis* protoplast cultures, has shown that inhibitors like the benzothiazole JZ1.24 promote cell reprogramming. These effects are associated with the modulation of BR-related genes and the induction of the somatic embryogenesis marker *SERK1*-like [10].

Complementary to kinase modulation, the regulation of cyclic nucleotide signaling (cNMPs) through PDE inhibition offers a strategy to sustain the intracellular lifespan of second messengers like cAMP and cGMP. These messengers function as essential hubs integrating environmental cues into developmental outputs [18,19]. The rationale for using PDE inhibitors originally characterized in mammalian models lies in the high evolutionary conservation of the PDE catalytic machinery. Early comparative analyses identified a conserved domain of approximately 200–270 amino acids shared across extremely diverse taxa, including mammals, yeast, and *Drosophila*, suggesting that the core mechanism for cNMP hydrolysis has been preserved throughout eukaryotic evolution [20]. Since PDEs function as metabolic “brakes” by terminating signaling cascades, their pharmacological inhibition could sensitize tissues to morphogenic stimuli, thereby enhancing regenerative efficiency. Positive effects of PDE inhibitors over the induction of cell reprograming have recently been observed in several plant in vitro systems [12].

Under the hypothesis that intervening in these signaling hubs can bypass endogenous developmental constraints, this study explores a preliminary phenotypic screening approach aimed at improving the in vitro performance of *Selenicereus* hybrids. Specifically, we aim to establish whether exogenous application of these compounds can elicit functional changes in tissue responsiveness, accelerating areole activation and increasing shoot yield, as observed with exogenous BRs in other *Cactaceae* [21,22]. By integrating inhibitors of GSK3, LRRK2, and PDEs, we seek to explore their capacity to sensitize tissues to morphogenic cues without compromising genomic stability. Ultimately, this study represents a first step toward integrating targeted chemical modulators into pitahaya tissue culture, serving as a phenotypic proof-of-concept. While the precise underlying biochemical pathways and potential off-target effects remain to be molecularly characterized, these inhibitors offer a new approach to complement traditional protocols, providing a foundational baseline to select the most promising candidates for future high-precision mechanistic studies.

## 2. Results

**Figure 1:**
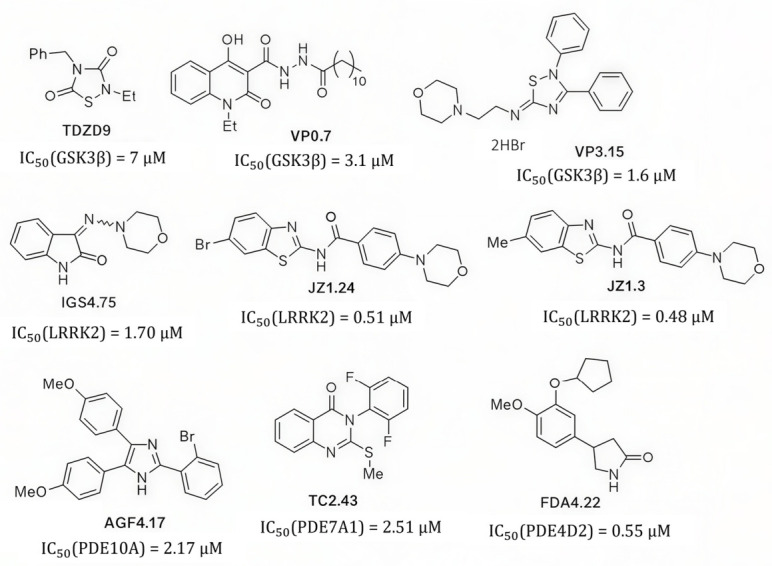
Kinase and phosphodiesterase inhibitors of mammals.

A variety of synthetic small molecules from our in-house Medicinal and Biological Chemistry (MBC) library [23] were selected for this study. These compounds, which belong to distinct chemical families, have previously been shown to increase the cell reprogramming rates in several in vitro plant cultures [10,11,12]. The selected molecules included the GSK3 inhibitors TDZD9, VP0.7 and VP3.15; the LRRK2 inhibitors JZ1.3, JZ1.24 and IGS4.75; and the PDE inhibitors TC2.43, FDA4.22 and AGF4.17 (Figure 1). In all cases, diverse chemical structures among the enzyme inhibitor class were selected. The concentration was established based on preliminary dose–response trials performed with this specific genotype, which indicated that 25 µM provided an optimal balance between morphogenic induction and explant viability, whereas higher concentrations previously reported in other systems (e.g., 50–100 µM) led to significant tissue necrosis in *Selenicereus* cladodes. All small molecules were evaluated in both PIT2 and a hormone-free basal medium (BM), given that many species within the *Selenicereus* genus can sprout without exogenous phytohormones once established in vitro. This dual approach enabled the characterization of morphogenic responses as a direct consequence of exposure to kinase and phosphodiesterase inhibitors across diverse physiological contexts.

### 2.1. Categorical Analysis of Morphogenic Response Types

**Figure 2:**
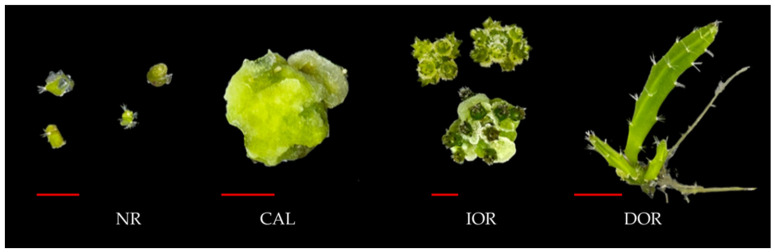
Categorization of morphogenesis types: no response (NR), callogenesis without differentiation (CAL), indirect organogenesis (IOR), and direct organogenesis/axillary budding (DOR). Scale bar = 10 mm.

The resulting morphogenic pathways were classified based on the primary developmental response (Figure 2): no response (NR), callogenesis (CAL), indirect organogenesis or axillary budding (IOR), and direct organogenesis (DOR).

**Figure 3:**
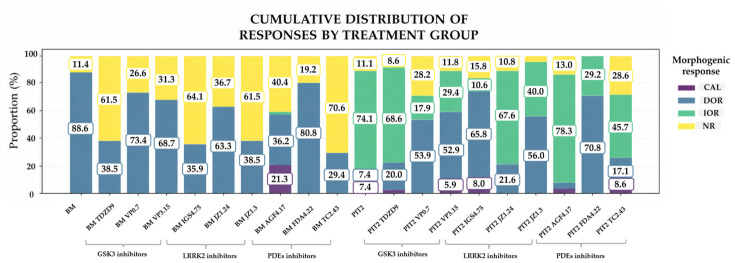
Proportional distribution of morphogenic response types in *Selenicereus* hybrid explants at day 75. The stacked bar chart illustrates the relative frequency (%) of each response category across treatments: callogenesis (CAL), direct organogenesis or axillary budding (DOR), indirect organogenesis (IOR), and no response (NR). Numerical values within each segment indicate the specific percentage for each category. Values < 5% are not shown; please refer to Supplementary Table S1 for complete data.

Categorical classification at day 75, that is, the time point at which the primary morphogenic pathways were fully established, revealed distinct distribution patterns among the treatments. These developmental trends are visually summarized in the stacked proportion plot (Figure 3), where a contrasting behavior is observed between treatments formulated on the basal medium (BM) and those using PIT2.

In basal treatments, the predominant response was direct meristematic budding (DOR), whereas indirect organogenesis (IOR) occurred at a substantially lower frequency, appearing notably only in the BM supplemented with AGF4.17 (approximately 21%). Furthermore, the addition of specific molecules was associated with a significant increase in the proportion of non-responsive explants (NR). This effect was particularly pronounced in treatments such as BM IGS4.75, BM JZ1.3, BM TDZD-9, and most sharply in BM TC2.43, where the NR category reached values as high as 70% of the explants.

In contrast, PIT2-based treatments exhibited a broader diversity of morphogenic outcomes. The PIT2 control (without molecules) showed a clear predominance of IOR, representing approximately 74% of the observed responses. A similar pattern was detected in several derived treatments, including PIT2 AGF4.17, PIT2 JZ1.24, PIT2 TC2.43, and PIT2 TDZD-9. However, other PIT2-based treatments displayed a shift toward DOR, most notably PIT2 FDA4.22 and PIT2 IGS4.75, where DOR frequencies reached approximately 71% and 66%, respectively (Figure 3, Supplementary Table S1).

Independence between treatments and response types was statistically confirmed via an χ^2^ test with Monte Carlo simulation, indicating that the distribution of morphogenic pathways was significantly dependent on the applied treatment (*p* < 0.001). To identify the specific treatment–response combinations contributing to this association, adjusted residuals were analyzed (Table 1).

In the basal group, significant positive deviations were observed for the DOR category across several treatments, while IOR showed systematic negative deviations. Conversely, PIT2-based treatments showed marked positive deviations for IOR, particularly in the control and those supplemented with AGF4.17, JZ1.24, and TDZD-9, accompanied by negative deviations for DOR.

**Figure 4:**
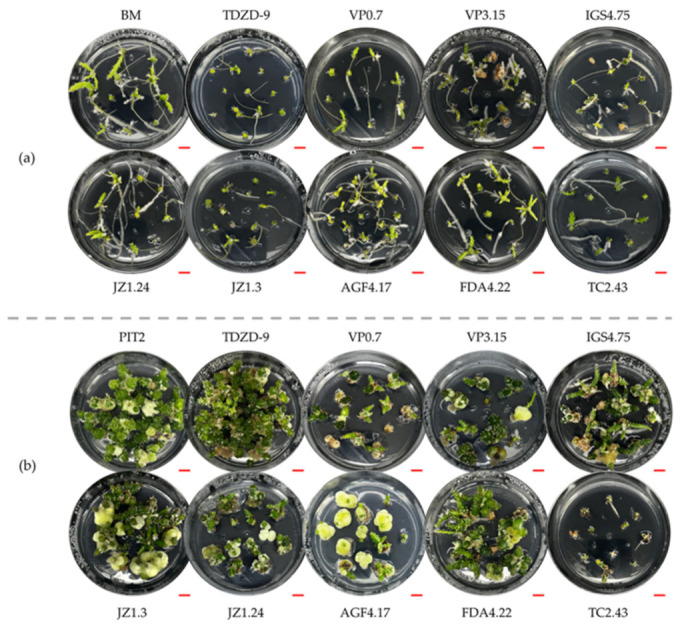
Representative examples of morphogenic diversity in pitahaya explants. Visual evidence of the diverse responses observed in (**a**) the basal medium and (**b**) hormone-supplemented PIT2 medium under different small-molecule treatments, in accordance with the patterns identified in the categorical analysis. Scale bar = 10 mm.

Pairwise post hoc contrasts with the Benjamini–Hochberg false discovery rate (FDR) adjustment further confirmed that significant differences (q_BH_ < 0.05) were primarily concentrated between the basal and PIT2 contexts. Within the basal group, AGF4.17 and TC2.43 stood out as the most differentiated treatments, the latter shifting significantly toward the NR category. Within the PIT2 group, FDA4.22 and IGS4.75 significantly altered the default morphogenic pattern by displacing the response from IOR toward DOR (Figure 4).

### 2.2. Quantitative Assessment of Shoot Yield

Beyond the distribution of response types, the intensity of regeneration was quantitatively assessed through the shoot yield (Y), considering only those explants that developed at least one shoot by day 75 (T2). Generalized linear models (GLMs) with a Poisson distribution and robust standard errors revealed a significant global effect of the treatment on Y (Robust Wald test, *p* < 0.001).

Early evaluation (T1, after 30 days of culture) showed fewer responding explants compared to T2, resulting in higher data dispersion and limited statistical power to detect formal differences at this stage. However, descriptively, PIT2-based treatments showed a higher frequency of responders and increased median values relative to BM at T1, characterizing an earlier response under growth regulator conditions. While inferential analysis focused on T2 when organogenic responses were fully established (Table 2), the temporal advantage observed at T1 was considered in the overall interpretation, as PIT2 treatments demonstrated increased precocity in shoot emergence.

At T2, BM presented a median of 3 shoots per explant (mean = 2.15; IQR: 1.00–3.00). Most supplemented basal treatments did not show consistent yield increases relative to the BM. Indeed, molecules such as JZ1.3, TDZD-9, and VP0.7 resulted in lower budding intensity, with means below 2 shoots per explant, placing them in the lower significance groups. Altogether, while the median yield for BM AGF4.17 (3 shoots) aligned with the control, individual events reached 10 shoots, surpassing the standard axillary capacity of 6 buds. However, this potential for additional budding did not yield a significant overall increase due to high internal variance.

In contrast, several PIT2-based treatments demonstrated superior performance. At T2, treatments including PIT2 TDZD-9, PIT2 TC2.43, PIT2 IGS4.75, and PIT2 JZ1.24 reached medians of 4 shoots per explant, positioning them in the top significance group. Other treatments, such as PIT2 FDA4.22 and PIT2 JZ1.3, showed intermediate values. Notably, the PIT2 control without molecules exhibited the lowest Y within its segment, with descriptive statistics similar to those of the basal medium. Despite the convergence observed at T2, PIT2-supplemented treatments exhibited superior yield metrics and faster response kinetics compared to non-supplemented media.

### 2.3. Morphological Quality Assessment of Regenerated Cladodes

In addition to the quantitative yield analysis, a qualitative assessment of the morphological appearance of regenerated shoots was conducted at day 75 to evaluate the structural quality of the material. Cladodes were classified into three categories based on their fidelity to standard ex vitro morphology: Category A (normal morphology similar to adult plants), Category B (flattened and partially hyperhydric morphology, but with the capacity to revert to normal growth), and Category C (severely distorted, rosette-like morphology with limited recovery potential).

**Figure 5:**
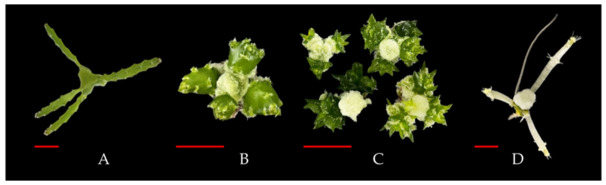
Classification of regenerated cladodes based on morphological quality: normal morphology (**A**), intermediate/reversible morphology (**B**), abnormal/rosette-like morphology (**C**), and etiolated and weakened morphology exhibiting loss of pigmentation prior to systematic necrosis (**D**). Scale bar = 10 mm.

All shoots obtained in BM, regardless of the presence of small molecules, initially exhibited Category A morphology (Figure 5).

However, a small fraction of these shoots (4.3% of the total BM explants) developed a weakened, etiolated appearance when maintained in culture beyond 60 days, occasionally showing pale or absent pigmentation, referred to as category D.

Unlike Category C shoots, which remained viable for extended periods, these etiolated shoots lost pigmentation and systematically progressed to necrosis without recovery. Consequently, as these represent exceptional and non-viable events (observed in only 11 out of 274 responding explants, exclusively within the hormone-free BM context), they were excluded from the final shoot yield counts and were not considered an independent morphological category. In contrast, PIT2-based treatments showed significantly higher morphological diversity, exhibiting a clear dependence on the specific molecule added. The interpretation of these patterns was based on the adjusted residuals from the contingency analysis (Table 3).

**Figure 6:**
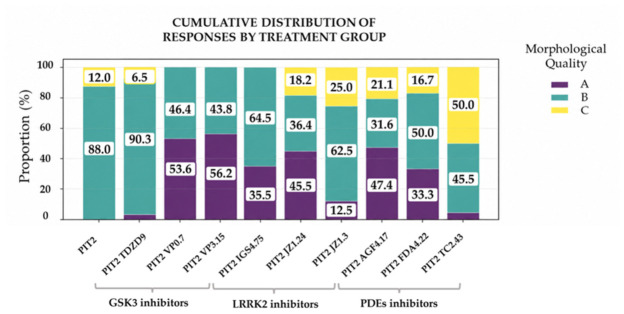
Proportional distribution of cladode morphological categories in *Selenicereus* hybrid explants across different culture media. The stacked bar chart illustrates the relative frequency (%) of each category for each treatment at day 75 where “A” represents normal morphology, “B” indicates flattened morphology with reversion capacity, and “C” denotes rosette-like morphology with limited recovery potential. Numerical values within the segments indicate the specific percentage for each category. Values < 5% are not shown.

The PIT2 control and PIT2 TDZD-9 treatments showed a strong positive association with Category B (residuals of 2.03 and 2.43, respectively) and negative associations with Category A. These specific media yielded a higher frequency of partially hyperhydric cladodes and a correspondingly lower proportion of normal shoots. Conversely, the addition of the molecules VP0.7 and VP3.15 led to a significant shift toward Category A (positive residuals of 2.49 and 2.08, respectively) and a reduction in distorted Category C forms. Unlike the BM-derived material, shoots that regenerated in these PIT2-supplemented media did not exhibit etiolation; instead, they maintained normal diameter, pigmentation, and high vigor throughout the experiment. Regarding undesirable morphologies, PIT2 TC2.43 showed the most marked positive association with Category C (4.45), with 50% of its explants producing severely deformed shoots (Figure 6). In contrast, PIT2 IGS4.75 showed a significant negative association with Category C (−2.10). While this treatment did not correlate with Category A morphology, it was associated with a reduction in the frequency of Category C developmental traits.

The global contingency analysis confirmed that cladode morphology was highly dependent on the culture medium (χ^2^ ≈ 89.5; Cramer’s V ≈ 0.42; *p* < 0.001). Post hoc comparisons with BH-FDR adjustment highlighted that the most significant shifts occurred in PIT2 VP0.7 and VP3.15, where Category A reached frequencies of 54% and 56%, respectively, significantly outperforming the PIT2 and PIT2 TDZD-9 treatments.

Overall, these results demonstrate that the combination of growth regulators with specific small molecules modulates not only the frequency of regeneration, but also the morphological quality of the resulting material. This allowed for a clear discrimination between treatments favoring viable clonal propagation and those inducing suboptimal morphological responses.

### 2.4. Integrated Evaluation: Global Efficiency Index (GEI) Ranking

The integration of the morphogenic response index, shoot yield, and morphological quality into the Global Efficiency Index (GEI), which was determined as described in the in Section 4, provided an indicative prioritization of the culture media based on their suitability for pitahaya multiplication. The top-performing treatments (led by PIT2 TDZD-9 and PIT2 FDA4.22) and the lowest-ranked groups (such as BM TC2.43 and BM TDZD-9) showed no significant shifts in position regardless of the coefficient distribution applied. In contrast, the intermediate positions exhibited greater oscillation depending on the specific weights assigned to the parameters. Notably, the relative positioning of BM and the PIT2 control fluctuated, with BM occasionally surpassing PIT2 when higher priority was given to morphological quality (M) or morphogenic response (R); the top-performing treatments (led by PIT2 TDZD-9 and PIT2 FDA4.22) and the lowest-ranked groups (such as BM TC2.43 and BM TDZD-9) showed no significant shifts in position regardless of the coefficient distribution applied. In contrast, the intermediate positions exhibited greater oscillation depending on the specific weights assigned to the parameters. Notably, the relative positioning of BM and the PIT2 control fluctuated, with BM occasionally surpassing PIT2 when higher priority was given to morphological quality (M) or morphogenic response (R), (Table 4). This holistic approach suggested that overall efficiency depends on a strategic interplay between response induction, shooting intensity, and the functional quality of the regenerated material.

The treatment achieving the highest GEI score was identified as PIT2 TDZD-9 (0.85), which excelled due to its high response rate (R_norm_ = 0.72) combined with the maximum productivity observed across all treatments (Y_norm_ = 1.00). Although this medium exhibited a moderate normalized morphological quality (M_norm_ = 0.62) due to a higher proportion of Category B cladodes, its exceptional performance in yield and response successfully compensated for this limitation. The second-positioned treatment was PIT2 FDA4.22 (GEI = 0.82), which showed the highest absolute response rate (R_norm_ = 1.00) alongside moderate yield and quality scores. In third place, PIT2 IGS4.75 (GEI = 0.72) was characterized by a balanced profile between response capacity (R_norm_ = 0.76), a moderate shoot yield (Y_norm_ = 0.68), and very high morphological quality (M_norm_ = 0.81).

Sensitivity analysis revealed that the relative distribution of the treatments remained consistent across the various explored weighting scenarios. The top-performing treatments (led by PIT2 TDZD-9 and PIT2 FDA4.22) and the lowest-ranked groups (such as BM TC2.43 and BM TDZD-9) showed no significant shifts in position regardless of the coefficient distribution applied. In contrast, the intermediate positions exhibited greater oscillation depending on the specific weights assigned to the parameters. Notably, the relative positioning of BM and the PIT2 control fluctuated, with BM occasionally surpassing PIT2 when higher priority was given to morphological quality (M) or morphogenic response (R).

As a consequence of the reduced active sample size in certain low-responding treatments, high intra-treatment variability led to an overlap in performance among these media, with no single culture medium simultaneously exhibiting the maximum values for both parameters. Therefore, the GEI combined these divergent response profiles into unified numerical trends, reflecting the multi-parametric distribution of the treatments.

### 2.5. Ex Vitro Acclimatization and Phenotypic Stability

Given the large number of regenerants obtained (>1500 individuals), the acclimatization phase was standardized by selecting 20 plants per treatment (*n* = 400). Priority was given to Category A and B plantlets, as they successfully underwent elongation and exhibited normal physiological behavior. Category C shoots were excluded from this phase due to their inability to elongate cladodes in the maturation medium (PitE).

During the elongation phase, temporal differences were recorded between morphotypes A and B. Category A shoots achieved a 100% elongation rate, reaching the required acclimatization size (10–15 cm in length) between days 85 and 97 of culture. Conversely, Category B shoots exhibited a wider developmental timeframe, with 95% of the shoots reaching the acclimatization stage between days 92 and 110. Within the total Category B regenerants (*n* = 664 shoots), 4.8% (32 shoots) arrested their growth and failed to elongate even after day 110. These 32 shoots were distributed uniformly across all treatments, and no association could be established between this growth arrest and any specific treatment due to its low incidence. The remaining viable Category A and B plantlets that were not selected for the acclimatization phase were subcultured and conserved in vitro to serve as a plant material bank for future experiments.

Out of the 400 selected plantlets, 394 successfully completed the hardening process, resulting in an overall survival rate of 98.5%. Occasional losses were primarily attributed to localized physiological stress related to substrate waterlogging. Acclimatized plants showed a steady establishment under ex vitro conditions, characterized by the emergence of new growth and a total absence of visible phenotypic aberrations.

**Figure 7:**
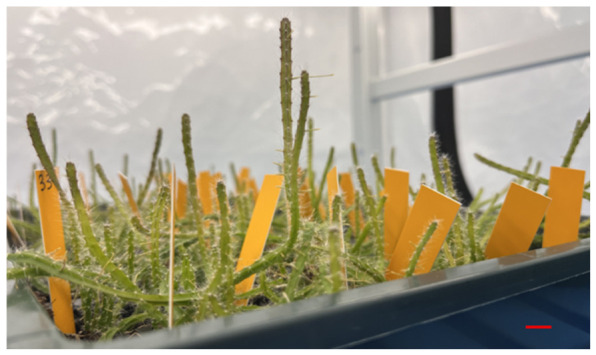
Morphological appearance and size of in vitro regenerated plants following the acclimatization phase (the number refers to the acclimatized plant identifier). Scale bar = 10 mm.

Upon full establishment, the plants exhibited homogenous morphological development, maintaining the typical architecture of the source genotype. No consistent differences were observed between treatments regarding cladode morphology or phenotypic stability, with minor variations limited to vegetative vigor (Figure 7). These results confirm that the in vitro application of kinase and phosphodiesterase inhibitors does not negatively impact the long-term viability or morphological integrity of *Selenicereus* hybrids.

### 2.6. Ploidy Level Stability Analysis

A subset of the acclimated plants was utilized to evaluate ploidy levels via flow cytometry. Ten plants were randomly selected from each treatment (representing 50% of the acclimated individuals per treatment) to detect cytogenetic stability associated with the different in vitro culture conditions and the exposure to small molecules.

All analyzed plants exhibited highly comparable fluorescence profiles, characterized by consistent G0/G1 peak positions and similar distributions of nuclei with higher relative DNA content. No significant peak shifts or additional nuclear populations, which would indicate aneuploidy or polyploidization, were detected across the various treatments. The observed profiles were compatible with physiological endopolyploidy, a phenomenon widely documented in *Cactaceae* vegetative tissues, rather than representing stable changes in the ploidy of the regenerated plants (Supplementary Table S2).

**Figure 8:**
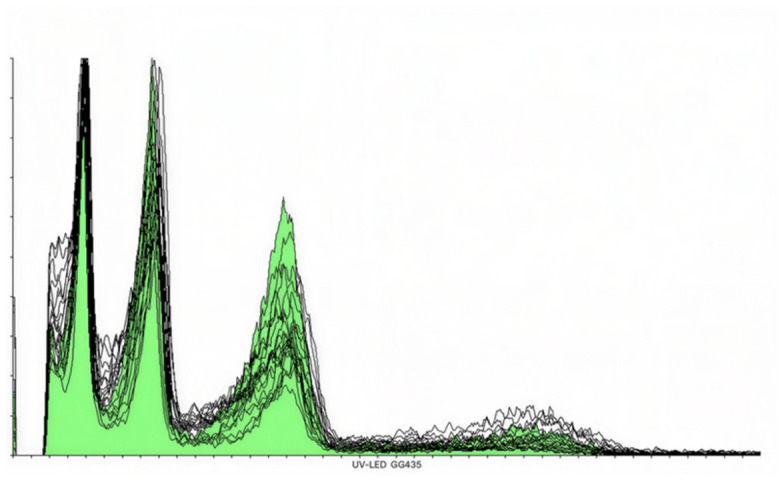
Flow cytometry histograms illustrating the relative DNA content and ploidy levels of the regenerated plants. The graph displays an overlay of 21 individual profiles: one representative plant from each of the 20 in vitro treatments, compared against the field-grown control material (same genotype, not subjected to in vitro culture), which is highlighted by the green shaded area.

Overall, the cytometric histograms of the in vitro regenerated and acclimated plants were indistinguishable from those obtained from field-grown control material of the same genotype. These results indicate that the established micropropagation protocol, including the application of kinase and phosphodiesterase inhibitors, maintains the nuclear DNA content stability of *Selenicereus* hybrids throughout the regeneration and acclimatization phases (Figure 8).

## 3. Discussion

The present study establishes a comprehensive framework for evaluating how targeted signaling modulation, via bioactive small molecules, influences the in vitro morphogenesis of *Selenicereus* hybrid explants. Unlike recent approaches primarily focused on somatic embryogenesis [10,11], our research assessed these chemical tools within the context of organogenesis and the activation of pre-existing meristems. Pitahaya was selected as a strategic model due to its high agricultural relevance and its physiological plasticity, characterized by an inherent capacity to sprout in minimal culture media [4]. This feature provided a unique opportunity to contrast the behavior of signaling inhibitors in a physiologically clean background versus a complex hormonal environment (PIT2). Our results reveal a profound interaction between the culture context and the chemical intervention: the effects observed in the hormone-free basal medium (BM) differed radically from those in the PIT2 medium across all qualitative and quantitative parameters, suggesting that the efficacy of these molecules is strictly gated by the endogenous signaling state of the explant.

A primary finding of this research was the molecules’ capacity to modulate or redirect the phenotypic outcome (direct or indirect morphogenesis) depending on the exogenous hormone applications. In the minimal BM system, which predominantly favors direct budding from meristems, most inhibitors exerted a repressive effect on bud activation, increasing the proportion of explants showing no response. Only a few of them maintained a profile similar to the control or slightly improved it without statistical significance. Conversely, in the PIT2 medium, where the baseline preference is indirect organogenesis, molecules such as IGS4.75 (LRRK2 inhibitor) and FDA4.22 (PDE inhibitor) significantly shifted the morphogenic default toward DOR while maintaining high production rates. This redirection is biologically significant for clonal fidelity, as direct regeneration from pre-existing meristems minimizes the risk of somaclonal variation associated with de-differentiated callus phases [24,25]. In fact, this aspect was integrated into the morphogenic response index (Rω) by assigning differentiated weights (ω = 1.00 for DOR vs. ω = 0.75 for IOR).

The performance of GSK3 inhibitors (TDZD-9, VP0.7, and VP3.15) provides a clear example of how the same enzymatic target can trigger divergent morphogenic outcomes depending on the “angle” of inhibition and the hormonal environment. While these molecules generally failed to enhance shoot yield in BM, they dominated the highest statistical significance groups in the PIT2 medium, with TDZD-9 reaching productivity levels far exceeding the control. Mechanistically, this high yield was intrinsically linked to the morphogenic pathway; TDZD-9 promoted the highest proportion of IOR responses, where the callus phase provides an expanded cellular surface for multiple budding sites, thus maximizing quantity. However, this productivity illustrates a “quantity–quality paradox”: the high yield often comes at the expense of structural integrity [26], as evidenced by the prevalence of Category B shoots, which demonstrated a delayed developmental pace during elongation. In contrast, other molecules within this family of inhibitors showed lower shoot yields but a higher affinity for direct pathways and Category A (normal) morphologies. From a molecular standpoint, the diversity in the results among these molecules is consistent with their distinct chemical structures and inhibitory strategies. In mammalian systems, these inhibitors target different regions of the kinase, employing distinct structural mechanisms such as allosteric modulation or specific residue binding [27]. However, mechanistic insights from mammalian models cannot be directly extrapolated to plant systems, and the precise cellular targets in *Selenicereus* remain to be elucidated. Our working hypothesis is based on the premise that structural and functional divergence among these compounds in mammals increases the probability of triggering distinct phenotypic responses in plant tissues. This approach does not assume conserved binding sites or pathways; indeed, the differential morphogenic outcomes could be largely driven by distinct off-target interactions or molecular promiscuity.

This perspective appears to be illustrated by the response pattern of the dual GSK-3β/PDE7 inhibitor VP3.15 in PIT2, which closely mirrored that of the selective GSK3 inhibitor VP0.7 rather than the specific PDE7 inhibitor TC2.43. This suggests that within the hypothetical context of pitahaya tissue culture, the morphogenic impact of VP3.15 might be predominantly driven by its interaction with putative plant GSK3 orthologs, while its dual-action capacity could remain secondary or absent. Consequently, such divergent behavioral profiles underscore the utility of these molecules as potential functional chemical probes, broadening the screening spectrum for tailoring specialized protocols in plant biotechnology.

A notable finding in this study was the contrasting behavior between GSK3 and LRRK2 inhibitors. While the former induced high-yield but often disorganized growth, LRRK2 inhibitors—particularly IGS4.75—promoted a more restrained yet morphologically superior response in PIT2 medium. The precise molecular basis for this consistency remains to be elucidated. Although LRRK2 inhibitors have been linked to the modulation of BR-related transcripts and cell reprogramming markers in other plant systems [10], our results do not yet allow for a definitive mechanistic conclusion. It is possible that the observed effects stem from a generalized stabilization of the regenerative niche rather than a specific signaling pathway. The fact that these molecules shifted the response toward direct organogenesis (DOR) while maintaining natural phyllotactic patterns suggests that they may act to stabilize developmental pathways in complex hormonal environments. However, given the multidomain nature of these proteins and the potential for cross-talk between signaling hubs, we cannot exclude the involvement of alternative, non-canonical pathways.

The PDE inhibitors yielded the most heterogeneous results among the families tested, a fact that underscores the complexity of cyclic nucleotide signaling in *Selenicereus*. While the use of these compounds was strategically based on the high evolutionary conservation of the PDE catalytic domain [20], it cannot be assumed that this structural homology necessarily ensures identical binding sites or affinities for mammalian-designed inhibitors in plant systems. The divergent morphogenic outcomes, ranging from the localized callogenesis induced by AGF4.17 in BM to the organized development (DOR) promoted by FDA4.22 in PIT2, suggest a nuanced signaling scenario. Furthermore, since the pharmacological action of PDE inhibitors is inherently dependent on the existence of an endogenous pool of cAMP or cGMP to ‘protect’, their efficacy may be strictly gated by the metabolic state of the explant at the time of treatment [19]. If these second messengers are not actively synthesized under our specific culture conditions, the observed phenotypes could instead stem from off-target interactions with non-canonical plant targets. Consequently, the severe rosette-like distortions (Category C) induced by TC2.43 or the shifts toward DOR by FDA4.22 should be interpreted as a phenotypic mapping of molecular potential rather than definitive proof of cNMP-mediated pathways. To resolve whether these molecules are effectively targeting second messenger turnover or acting via off-target mechanisms, future research must specifically quantify intracellular cNMP fluctuations during the culture period and utilize RNA sequencing to identify the underlying transcriptional changes.

Because each analyzed parameter provides only a partial view of treatment success, the implementation of the GEI offers a practical approach for a more integrated assessment. The GEI assisted in weighing the trade-offs yield and quality, identifying PIT2 TDZD-9 and PIT2 FDA4.22 as the most prominent treatments within this screening. While TDZD-9 maximizes sheer productivity, FDA4.22 offers a superior balance by combining high yield with a shift toward direct organogenesis, providing a more robust framework for high-quality propagation. This analytical framework served as a useful interpretive tool, facilitating the detection of general trends between treatments and providing a clear, normalized basis for decision-making at this exploratory stage in plant biotechnology.

Finally, the cytogenetic stability and acclimatization results validate the safety of this targeted signaling modulation. The maintenance of nuclear DNA content, confirmed by flow cytometry, suggests that these inhibitors—even those acting on complex kinase pathways—do not induce large-scale genomic instability such as aneuploidy [7,28]. Although subtle epigenetic modifications cannot be excluded [29], the 98.5% survival rate and the phenotypic uniformity of the acclimatized plants (Figure 7) provide strong evidence for the practical viability of these optimized chemical interventions in *Selenicereus* breeding and conservation programs.

While our phenotypic observations provide a solid baseline, interpreting these responses requires looking at the established literature in model plants. For instance, the inhibition of GSK3-like kinases is known to positively regulate brassinosteroid signaling, which extensively crosstalks with pathways driving organogenesis. We hypothesize that molecules like TDZD-9 and IGS4.75 might induce the observed morphogenic shifts in *Selenicereus* through a similar pathway derepression. However, because our data are strictly phenotypic and these modulators were designed for mammalian targets, such mechanistic extrapolations must be treated with caution. Future transcriptomic and phosphoproteomic analyses are strictly required to confirm the exact molecular targets and determine whether the observed outcomes stem from the intended inhibition or potential off-target effects. From a methodological standpoint, the utility of integrating multi-parametric datasets suggests that our weighting framework could be adapted to other recalcitrant cactus species where balancing yield and quality is difficult. Ultimately, long-term field monitoring of the regenerated plants remains essential to evaluate whether these targeted chemical interventions exert subtle effects on fruit quality, precocity, or multi-year phenotypic stability, thereby ensuring commercial viability.

## 4. Materials and Methods

### 4.1. Plant Material

The experiments were conducted using a commercially significant interspecific hybrid derived from the hybridization between *Selenicereus guatemalensis* and *Selenicereus undatus*. Plant material was sourced from mother plants established at ‘El Bardo’ experimental station (Beyond Seeds Group; 36.818131, −2.235117) located in Cabo de Gata (Almería, Spain). Explant collection and subsequent in vitro initiation were performed during the active vegetative growth period (March–September).

### 4.2. Morphogenesis Induction and Culture Conditions

For the induction assays, transverse sections (<1 cm) were excised from in vitro-derived cladodes obtained through the sprouting of areolar meristems. To avoid hormonal carry-over and physiological aging, cladodes were sourced exclusively from PGR-free media with fewer than three subcultures. These donor tissues were further restricted to those with fewer than three previous subcultures and a standardized length between 7 and 10 cm. For each experimental treatment, five independent Petri dishes were used as replicates, each containing nine explants (*n* = 45 per treatment).

### 4.3. Culture Media and Experimental Context

Two contrasting physiological environments were established to evaluate the impact of the chemical modulators. All culture media were prepared using Murashige and Skoog (MS) basal salts and vitamins (M0222, Duchefa Biochemie, Haarlem, The Netherlands), supplemented with 30 g L^−1^ sucrose and solidified with 5 g L^−1^ Gelrite (G1101, Duchefa Biochemie). For hormonal treatments, 6-benzylaminopurine (BAP; B0904, Duchefa Biochemie) and indole-3-butyric acid (IBA; I0902, Duchefa Biochemie) were added according to the experimental design (Table 5). The pH was adjusted to 5.7 ± 0.1 using NaOH or HCl prior to sterilization.

Media were sterilized by autoclaving at 121 °C for 20 min. Under aseptic conditions, approximately 38 mL of medium was dispensed into deep sterile Petri dishes (90 × 25 mm). Prepared media were stored at room temperature and used within three days of preparation. All cultures were incubated in a growth chamber at 24 ± 2 °C under a 16-h photoperiod. Illumination was provided by cool-white, fluorescent lamps with a photosynthetic photon flux density (PPFD) of 50 µmol m^−2^ s^−1^.

### 4.4. Small Molecule Library and Treatment Preparation

To explore novel morphogenic regulation strategies, nine bioactive small molecules were selected based on their pharmacological relevance: three GSK3 inhibitors (TDZD-9, VP3.15, and VP0.7), three LRRK2 inhibitors (JZ1.24, JZ1.3, and IGS4.75), and three phosphodiesterase (PDE) inhibitors targeting PDE4, PDE7, and PDE10 (FDA4.22, TC2.43, and AGF4.17, respectively). FDA4.22 (Rolipram) was acquired from commercial sources. The rest of the compounds were synthesized in our laboratory at the CIB-CSIC following previously described procedures [12,27,30].

Stock solutions (10 mM) were prepared in molecular biology grade dimethyl sulfoxide (DMSO), sterilized via membrane filtration (0.22 µm), and stored at −20 °C in light-protected aliquots. The inhibitors were added to the culture media after autoclaving once the medium reached approximately 50–55 °C. A final working concentration of 25 µM was selected for all chemical treatments. Control treatments (BM and PIT2 without inhibitors) were supplemented with an equivalent volume of DMSO (0.25% *v*/*v*), while an additional baseline control devoid of DMSO (0%) was run concurrently to confirm the absence of solvent-induced artifacts (Supplementary Table S3). Consequently, the complete experimental design encompassed a total of 20 treatments, in addition to the two supplementary baseline media used to rule out any solvent influence.

### 4.5. Shoot Maturation, Elongation, and Acclimatization

Following the initial 30-day induction period, explants were subcultured onto a maturation and elongation medium (PitE, MS medium supplemented with 0.5 mg/L IBA) for an additional 45 days, during which the small-molecule inhibitors were omitted.

Acclimatization was initiated progressively as individual regenerated shoots achieved the standard developmental threshold, characterized by a primary cladode length of 10–15 cm and the presence of functional roots. Consequently, the transplantation phase was staggered between day 85 and day 110 of total culture, depending on the individual growth kinetics of the shoots. Prior to transplantation, plantlets were thoroughly washed with distilled water to remove any residual gelling agent from the root system. Then, plantlets were transplanted into 30-cell plastic trays housed inside propagators equipped with transparent vented domes. The trays contained a commercial cactus-specific substrate (COMPO SANA^®^ Cactus; COMPO GmbH, Münster, Germany) composed of high-quality peat, quartz sand, and pumice, enriched with a slow-release NPK (14-16-18) fertilizer. Substrate irrigation was standardized by applying 20 mL of water per cell every 48 h using low conductivity tap water (≈200 µS cm^−1^), administered dropwise, complemented by distilled water misting on the tray lids to maintain high relative humidity during the first stage of hardening. After 24 h, the lid vents were partially opened to allow for gradual humidity reduction, and the covers were completely removed by the fifth day. Trays were maintained in a growth chamber at 25 ± 2 °C under a 16-h photoperiod.

### 4.6. Ploidy Level Determination via Flow Cytometry

Once the regenerated plants were established ex vitro, their ploidy levels were assessed and compared against control material from field-grown adult plants to ensure cytological stability. Relative nuclear DNA content was determined by flow cytometry following the method described by Doležel et al. [31], with specific modifications to mitigate the interference of high mucilage content typical of *Cactaceae* tissues.

Fresh cladode segments (2–3 cm) were used for each analysis. Nuclei were released by mechanical dissociation in cold LB01 extraction buffer. For field-grown material, the homogenate was incubated at 4 °C for 2–3 h to facilitate the sedimentation of tissue debris and mucilaginous compounds. The supernatant was then carefully recovered and filtered through a 30 µm nylon mesh. In contrast, in vitro-derived material, characterized by lower lignification, underwent a less intensive mechanical dissociation followed by immediate filtration.

Following filtration, an equal volume of staining solution (CyStain™ UV Precise P; Sysmex, Hamburg, Germany) was added, and samples were incubated in the dark for 10 min. Nuclear fluorescence was analyzed using a CyFlow^®^ Space flow cytometer (Sysmex) equipped with a UV excitation source. The gain was fixed at 545 to record the G0/G1 phase peaks. Ploidy stability was confirmed by comparing the relative positions of the G0/G1 peaks between in vitro regenerated samples and the field-grown controls.

### 4.7. Data Collection and Morphogenic Assessment

Data collection was conducted at two specific stages: at the end of the induction phase (day 30) and upon completion of the maturation period (day 75). Observations at day 30 were restricted to the counting of incipient shoots to monitor early organogenic activation.

The full characterization of the results was performed at day 75, where qualitative morphogenic response types were recorded by categorizing explants into callogenesis (CAL), indirect organogenesis (IOR), direct organogenesis including axillary budding (DOR), or no response (NR), (Figure 2). Simultaneously, the functional morphology of the regenerated material was assessed and classified into four distinct groups consisting of normal cladodes (A) exhibiting standard morphology similar to field-grown plants, intermediate tissues (B) representing flattened or slightly hyperhydric cladodes capable of reverting to normal growth after subculturing, abnormal structures (C) consisting of severely distorted, rosette-like morphology with limited recovery potential and etiolated and weakened morphology (D) exhibiting loss of pigmentation (Figure 5).

Quantitative data regarding the number of shoots per explant were consolidated at both time points to evaluate the shoot yield (Y) and its temporal evolution. Furthermore, the exact calendar date when each individual plantlet met the developmental thresholds and was transferred to soil was systematically recorded. This allowed for the precise calculation of the elongation period required for each shoot to reach full readiness for ex vitro acclimatization.

To ensure high procedural rigor and eliminate potential observer bias, a blind assessment was conducted where each Petri dish was identified with a random numerical code. All experimental data, such as culture media composition, were managed through the NOAH-ERP v2.0.19388 database (Bullsoft Solutions S.L.), while systematic photographic documentation of all replicates was performed.

### 4.8. Statistical Analysis

The explant was defined as the primary observational unit. To account for potential intra-plate dependence (e.g., shared microenvironmental conditions or gas exchange), the Petri dish was incorporated as a clustering unit in the inferential models.

#### 4.8.1. Categorical Data Analysis

The association between treatments and morphogenic/morphological categories was assessed using contingency tables. Global associations were evaluated via the χ^2^ test or Fisher’s exact test with Monte Carlo simulation (10^4^–10^5^ permutations) for tables with low expected frequencies. Standardized adjusted residuals were inspected to identify specific categories contributing to significant associations, and *p*-values were adjusted using the Benjamini–Hochberg method to control the false discovery rate (FDR).

#### 4.8.2. Quantitative Data Analysis

The number of shoots was analyzed as a count variable. Due to the presence of non-responding explants, a two-step modeling approach was adopted, where shoot intensity was analyzed conditioned on the occurrence of an organogenic response. Generalized linear models (GLMs) with a Poisson distribution and a log-link function were fitted. Robust standard errors clustered by plate were used to ensure conservative inference. The global treatment effect was assessed using a robust Wald test. Pairwise comparisons were performed through linear contrasts, with *p*-values adjusted using Holm’s method. Statistical analyses were implemented in Python (v. 3.11) using pandas, numpy, scipy, statsmodels, and scikit-posthocs. Visualizations were generated with matplotlib and seaborn.

### 4.9. Integrated Evaluation: Global Efficiency Index (GEI)

A Global Efficiency Index (GEI) was developed by integrating three critical dimensions derived from the previously described observations. These dimensions consist of a weighted morphogenic response index (Rω), the shoot yield (Y), and a weighted morphological quality index (Mω).

The first component, the weighted morphogenic response index Rω, was calculated based on the relative proportion of each morphogenic response type according to biological desirability where DOR was assigned a weight of 1.00; IOR a weight of 0.75; CAL a weight of 0.10; and NR a weight of 0.00. The second component, Y, was estimated as the average number of shoots produced per responding explant in each treatment. Finally, the weighted morphological quality index (Mω) was determined by the proportion of morphological appearance categories assigned according to functional relevance with normal cladodes (Type A) weighted at 1.0, intermediate tissues (Type B) at 0.8, and abnormal structures (Type C) at 0.0. Morphology Type D was excluded from the analysis due to its negligible incidence. All variables were normalized using a Min–Max transformation to a 0–1 range (Equation (1)).

X_norm_ = (X − X_min_)/(X_max_ − X_min_) (1)

The final GEI was then calculated as a weighted combination of these normalized values where weights of ω_1_ = 0.4, ω_2_ = 0.5, and ω_3_ = 0.1 were assigned to the normalized parameters: R_norm_, Y_norm_, and M_norm_, respectively (Equation (2)).

GEI = (ω_1_·R_norm_) + (ω_2_·Y_norm_) + (ω_3_·M_norm_) (2)

A sensitivity analysis was performed by varying the selection of coefficients using alternative weighting schemes. These variations were restricted to biologically plausible ranges while preserving the model’s core architecture to evaluate the consistency of the resulting treatment rankings.

## 5. Conclusions

The present study demonstrates that optimizing micropropagation protocols for pitahaya requires an integrative approach that simultaneously balances explant responsiveness, shoot yield, and morphological quality. In this framework, the Global Efficiency Index (GEI) was utilized as a convenient mathematical framework for synthesizing complex, multi-parametric data, helping to avoid partial interpretations based on isolated variables. Our findings reveal that the functional efficacy of these small-molecule signaling modulators is critically dependent on the physiological context of the explant. While putative kinase and phosphodiesterase inhibitors generally repressed budding in hormone-free media, they acted as potent amplifiers of productivity when combined with exogenous growth regulators. Specifically, the combination of the PIT2 medium with the GSK3 inhibitor TDZD-9 emerged as the most efficient treatment within this screening, successfully maximizing shoot yield (Y) while maintaining acceptable morphological quality (M).

Furthermore, this work highlights the potential of specific chemical families to refine the quality of the regenerated material. While GSK3 inhibitors prioritized quantitative yield, the use of LRRK2 inhibitors such as IGS4.75 proved effective in preserving architectural integrity and eliminating developmental distortions induced by high-cytokinin environments. The inclusion of morphological categories within the GEI allowed for a balanced selection that ensures both high productivity and functional viability.

Finally, the conservation of ploidy levels and the high survival rates during acclimatization confirm that these chemical interventions do not compromise the cytogenetic stability or the ex vitro performance of *Selenicereus* hybrids.

In conclusion, our results highlight the considerable potential of these innovative small molecules, originally designed as inhibitors of mammalian kinases and PDEs, as novel tools for improving plant micropropagation systems and expanding the range of strategies available beyond traditional plant growth regulators.

## Figures and Tables

**Table 1:**
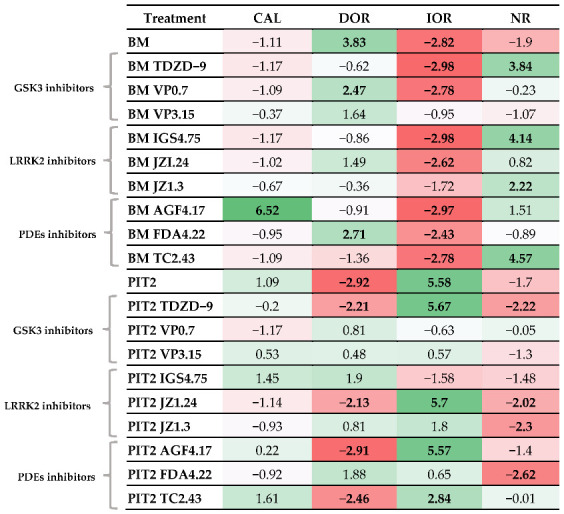
Adjusted residuals from the contingency analysis (Treatments × Morphogenic Response Types) in *Selenicereus* hybrid explants. Statistically significant values (|AR| ≥ 2) in bold. A heatmap color scale has been applied to the background to aid in data interpretation. The gradient highlights positive values in green and negative values in red.

**Table 2:**
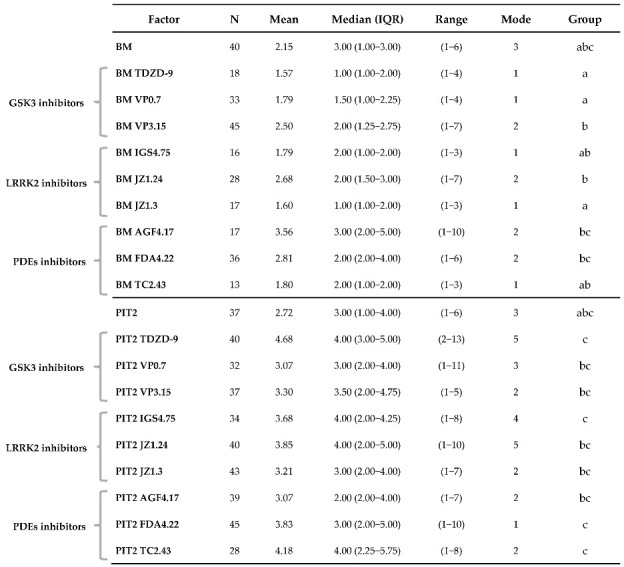
Shoot yield (Y) assessment (number of shoots per responding explant) at evaluation time T2. Multiple comparisons are based on GLM (Poisson) with robust standard errors clustered by plate. Different lowercase letters indicate significant differences between treatments (*p* < 0.05).

**Table 3:**
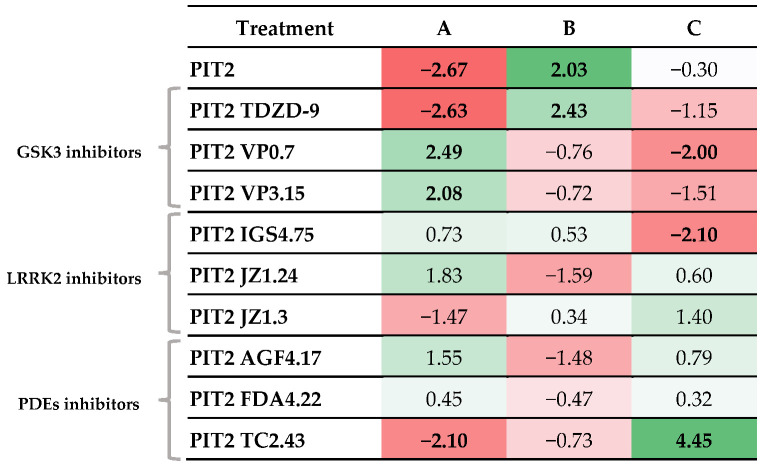
Adjusted residuals from the contingency analysis (Treatments × Morphological Quality) in *Selenicereus* hybrid explants. Statistically significant values (|AR| ≥ 2) are indicated in bold. A heatmap color scale has been applied to the background to aid in data interpretation. The gradient highlights positive values in green and negative values in red.

**Table 4:** Integrated assessment of morphogenic response (R), shoot yield (Y), and morphological quality (M) with their respective normalized values and the final Global Efficiency Index (GEI).

Treatment	R	Y	M	$R_{\mathrm{norm}}$	$Y_{\mathrm{norm}}$	$M_{\mathrm{norm}}$	GEI ^1^
PIT2 TDZD-9	0.72	4.68	0.74	0.72	1.00	0.62	0.85
PIT2 FDA4.22	0.93	3.83	0.70	1.00	0.73	0.57	0.82
PIT2 IGS4.75	0.74	3.68	0.87	0.76	0.68	0.81	0.72
PIT2 JZ1.24	0.72	3.85	0.71	0.73	0.73	0.58	0.72
PIT2 VP3.15	0.76	3.30	0.91	0.77	0.56	0.87	0.68
PIT2 JZ1.3	0.86	3.21	0.58	0.91	0.53	0.39	0.67
BM FDA4.22	0.81	2.81	1.00	0.84	0.40	1.00	0.64
PIT2 TC2.43	0.52	4.18	0.31	0.47	0.84	0.00	0.61
PIT2 VP0.7	0.67	3.07	0.91	0.67	0.48	0.87	0.59
BM	0.89	2.15	1.00	0.95	0.19	1.00	0.57
BM AGF4.17	0.40	3.56	1.00	0.31	0.64	1.00	0.54
PIT2 AGF4.17	0.63	3.07	0.68	0.62	0.48	0.54	0.54
BM JZ1.24	0.63	2.68	1.00	0.61	0.36	1.00	0.52
PIT2	0.64	2.72	0.68	0.62	0.37	0.54	0.49
BM VP0.7	0.74	1.79	1.00	0.75	0.07	1.00	0.43
BM VP3.15	0.17	2.50	1.00	0.00	0.30	1.00	0.25
BM IGS4.75	0.36	1.79	1.00	0.25	0.07	1.00	0.24
BM JZ1.3	0.38	1.60	1.00	0.29	0.01	1.00	0.22
BM TDZD−9	0.38	1.57	1.00	0.29	0.00	1.00	0.21
BM TC2.43	0.29	1.80	1.00	0.17	0.07	1.00	0.20

^1^ GEI was calculated as a weighted combination of normalized parameters: GEI = (ω_1_·Rnorm) + (ω_2_·Ynorm) + (ω_3_·Mnorm), where ω_1_ = 0.4, ω_2_ = 0.5, and ω_3_ = 0.1.

**Table 5:** Composition of the culture media used for pitahaya morphogenesis induction.

Components	PIT2	BM
MS	4.4 g/L	4.4 g/L
Sucrose	30 g/L	30 g/L
6-BAP	5 mg/L	-
IBA	0.3 mg/L	-
Gelrite	5 g/L	5 g/L

## Data Availability

All data are presented within the article.

## Supplementary Materials

The following supporting information can be downloaded at: https://www.mdpi.com/article/10.3390/plants15131931/s1, Table S1: Proportion of morphogenic response types in Selenicereus hybrid explants at day 75 for each treatment; Table S2: Mean channel position of detected peaks and G0/G1 coefficient of variation (CV) under different treatments; Table S3: Morphogenic response type, shoot yield, and morphological quality of control groups (DMSO vs. non-DMSO).

## Abbreviations

The following abbreviations are used in this manuscript:

6-BAP	6-Benzylaminopurine
AGF4.17	Imidazole-like PDE10A inhibitor
AR	Adjusted residuals
BH-FDR	Benjamini–Hochberg false discovery rate
BIN2	Brassinosteroid-Insensitive 2
BM	Basal medium
BR	Brassinosteroid
CAL	Callogenesis
cAMP	Cyclic adenosine monophosphate
cGMP	Cyclic guanosine monophosphate
CIB-CSIC	Margarita Salas Center of Biological Research
cNMPs	Cyclic nucleotides monophosphate
DOR	Direct organogenesis
FDA4.22	Rolipram (Commercial PDE4 inhibitor)
GEI	Global Efficiency Index
GLMs	Generalized linear models
GSK3	Glycogen synthase kinase 3
IBA	Indole-3-butyric acid
IGS4.75	Indolinone-based LRRK2 inhibitor
IOR	Indirect organogenesis
IQR	Interquartile range
JZ1.24	Benzothiazole-based LRRK2 inhibitor
JZ1.3	Benzothiazole-based LRRK2 inhibitor
LRRK2	Leucine rich repeat kinase 2
MBC library	Medicinal and Biological Chemistry library
Mnorm	Normalized morphological quality
NR	No response
PDE	Phosphodiesterase
PGRs	Plant growth regulators
PIT2	Plant growth regulator-supplemented medium
PitE	Elongation medium
TC2.43	Thioxoquinazoline-based PDE7 inhibitor
TDZD9	Thiadiazolidinone-9 (GSK3 inhibitor)
VP0.7	Allosteric GSK3 inhibitor
VP3.15	Dual GSK-3β/PDE7 inhibitor
Y	Shoot yield
Ynorm	Normalized shoot yield
